# Extrapulmonary Sarcoidosis Presenting as Bilateral Lower Limb Erythema Nodosum Triggered by Intradermal Fillers: A Case of Misdiagnosis As Soft-Tissue Infection

**DOI:** 10.7759/cureus.75220

**Published:** 2024-12-06

**Authors:** Alice Musher, Melanie S Diaz Ortiz, Antonio Diaz Lizarraga

**Affiliations:** 1 Department of Medicine, MetroWest Medical Center, Framingham, USA

**Keywords:** cosmetic procedures, dermal filler, erythema nodosum, extra pulmonary manifestations of sarcoidosis, panniculitis

## Abstract

Localized inflammatory reactions in patients with past procedural history of intradermal injections can quickly drive the clinician's attention towards a diagnosis of soft-tissue infection in the context of symptoms such as fever, malaise, and local induration of the adipose panniculus. However, in patients with a long-term history of granulomatous events, a rheumatologic approach must be taken into consideration when the clinical course overwhelms the odds for more conventional diagnoses. In this case, a 39-year-old female patient who underwent bilateral lower limbs intradermal filllers presented with a two-year clinical course of repetitive flares of external bilateral hip tenderness, pain that limits her walking, soft-tissue nodular inflammation, redness, fever and a soft mobile nonpainful right supraclavicular lymphadenopathy. She sought medical attention at the Emergency Department in a community hospital of Framingham, Massachusetts, due to the aggravation of a repeated clinical episode. With a former diagnosis of recurrent bilateral soft-tissue infection, the patient underwent a novel approach different from the suspicion of a resistant pathogen towards the scope of a reminiscent granulomatous condition triggered by the two-year-old cosmetic para gluteal injections. After treatment with a course of systemic steroids and a deeper interrogation of past medical history, her clinical evolution highlighted the importance of seeking appropriate medical counseling prior undergoing cosmetic procedures. This is particularly critical for patients with a history of sarcoidosis, a granulomatous condition that can be flared even decades after the initial diagnosis when it meets specific triggers, such as intradermal injection. This risk is potentially heightened when such procedures are performed by underqualified providers who may lack the skill to gather a comprehensive medical history or, even worse, execute inadequate techniques or work in suboptimal environmental conditions, thereby increasing the potential for harm.

## Introduction

The increasing popularity that intradermal procedures have gained in the aesthetic world has been attracting a wide variety of patients for the rapid and accessible, minimal-invasive techniques that pledge instant and durable results. However, these procedures have the potential to cause detrimental effects in carelessly selected patients and/or when procedures are not performed by a qualified professional with the appropriate dexterity and resources.

T-cell delayed type IV hypersensitivity reaction from the immune system against external antigens, such as dermal fillers, have the potential to cause inflammatory granulomatous conditions in susceptible patients [1]. For this reason, cosmetic dermal fillers are not generally recommended for patients with sarcoidosis. Nevertheless, there has been an alarming increase in non-traditional cosmetic “med spas” where procedures of variable invasion are being performed without adequate medical oversight. Erythema nodosum, a type of painful panniculitis that forms part of the spectrum presentation of sarcoidosis, resembles soft-tissue infection that might potentially lead to misdiagnosis or delay an accurate diagnosis in patients with old medical history of sarcoidosis [1]. Many times, patients might even have forgotten receiving such a diagnosis and not only they struggle to connect it with an active flare but also they disarray the process of medical interview by not providing or delaying to provide clinical history that can have an impact on the success of initial approaches in clinical judgment, which also is a reminder of the artistic process involved in conducting a fruitful patient assessment.

## Case presentation

A 39-year-old female patient with recurrent flares of signs and symptoms of acute-on-chronic inflammatory process has been seen twice in the past by another nearby medical center in which she received the same diagnosis of bilateral cellulitis and, at a clinician, discrete antibiotic treatment; supplementary to this, patient has self-treated with as needed thermo-/cryo-therapy (alternating warm and ice packs locally) and daily non-steroidal anti-inflammatory drugs (NSAIDs). At the time of hospital admission during her most recent presentation of symptoms, the patient revealed that the first onset of inflammatory symptoms occurred a year ago, one year after receiving an unknown dermal filler on both external hips for aesthetic reasons at a local med spa that curiously is now closed to the public. During the current episode, she reported the same gradual clinical evolution of symptoms, starting two days prior to hospital admission with increasing swelling, redness, and pain on the upper external area of both lower limbs, gradual elevation of temperature levels (highest recorded at home was 101°F) and malaise. At the time of triaging, the patient-relevant physical findings were a temperature of 100.6°F, blood pressure (BP), 122/71 mmHg; heart rate (HR), 121 bpm, respiratory rate (RR), and 18 rpm. Pain was catalogued as 10/10 on the pain scale. The patient denied recent travel or any other relevant personal, family, or social medical history at the time of triaging. She was subsequently admitted under the presumptive diagnosis of a soft-tissue infection, and intravenous analgesics were started with empiric antibiotic treatment after sampling for laboratory tests.

In the following days, the patient showed inexistent improvement on the clinical status and investigation for the differential diagnosis led the clinical team to re-interview the patient for cues toward a rheumatologic approach. During questioning, the patient claimed receiving a sarcoidosis diagnosis during her early youth due to a dermatologic lesion on one foot. Since the patient is Brazilian and this diagnosis was made many years ago in her country of origin, she has no reliable documentation at hand to support her diagnosis and has no memory of any other relevant aspect related to it. Given former events, a case of sarcoidosis manifested as bilateral erythema nodosum (EN) started to be considered; motion that was enhanced after consultation with the Infectious disease department, which ruled out a potential source of infection as the origin for the recurrent clinical picture of the patient.

Upon physical exam, the patient was in evident distress due to pain in lower limbs, she also expressed slight lower abdomen discomfort. Other than that, she was cooperative, oriented in person, time, and place.

Regarding the positive relevant findings in anatomical order, there was a non-tender, mobile, swollen lymph node occupying an area of approximately 1.5 inches on the left supraclavicular bone. Lymphadenopathy was characterized by a regular and oval shape, with a soft consistency at palpation.

**Figure 1:**
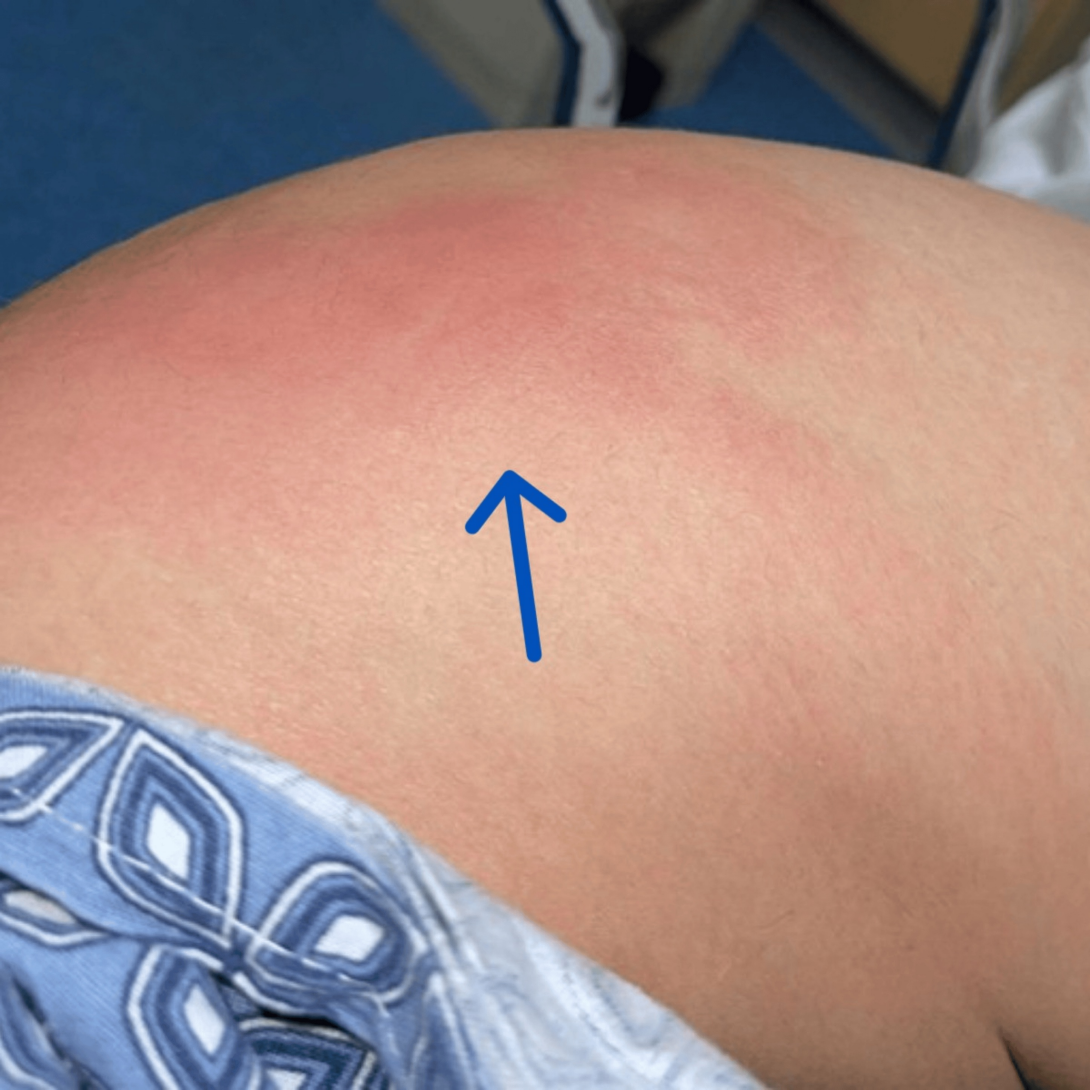
Right hip. Blue arrow pointing at the localized area exhibiting inflammatory signs.

The examination of the right lower extremity revealed a swelled, indurated, tender to palpation, and warmth area of approximately 3 inches (Figure 1).

**Figure 2:**
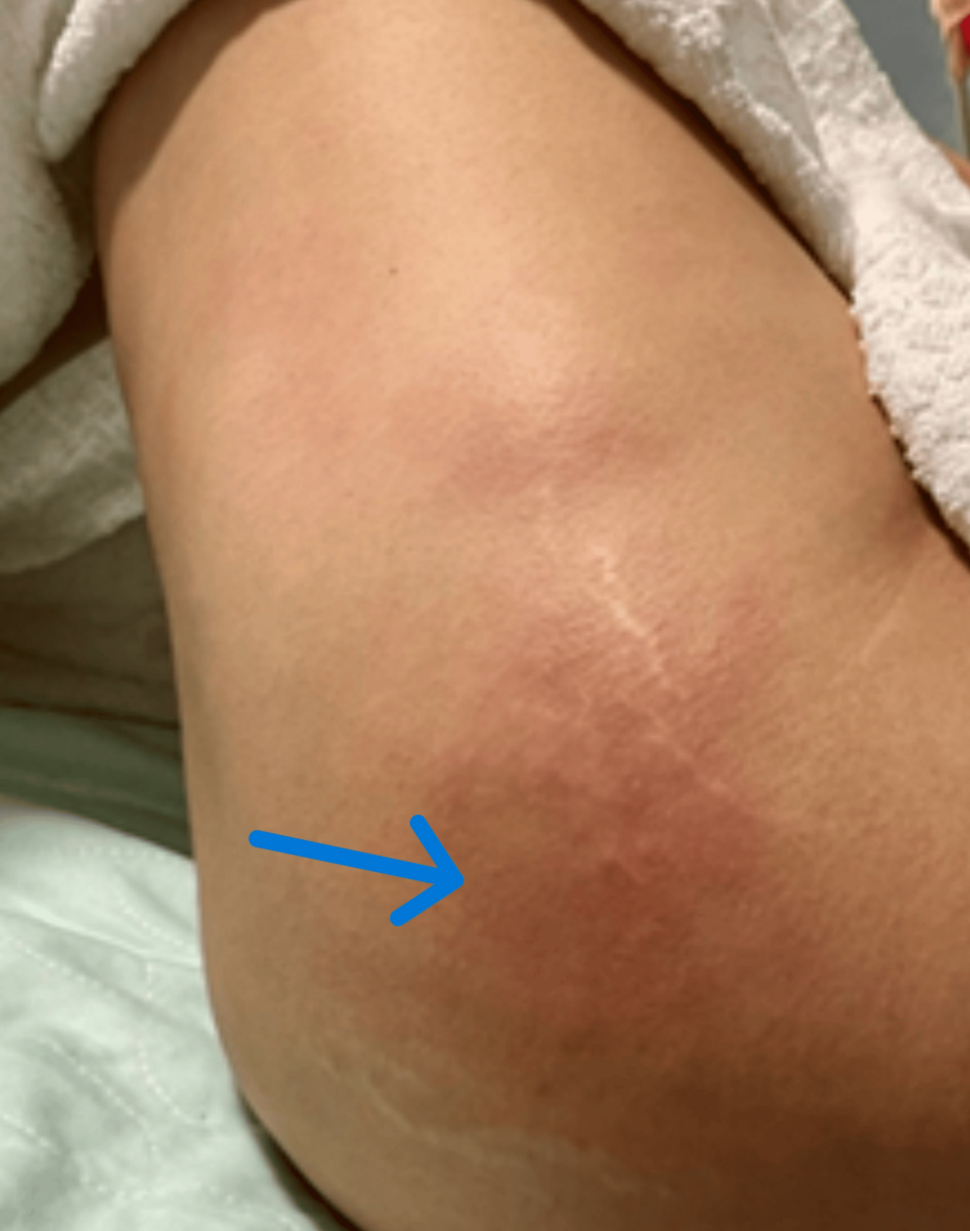
Left hip. Blue arrow pointing at the localized area exhibiting inflammatory signs.

On the lateral aspect of the left hip, an inflamed area measuring 4-5 inches was observed, exhibiting an identical description from the contralateral hip (Figure 2).

There was no skin breakdown, streaking, crepitus or discharge in any of the lesions encountered. At passive maneuvers, the patient had full range of motion (ROM) of both hips, but severe pain upon external rotation of left hip and left knee flexion. Gait evaluation was deferred due to pain.

Pertinent laboratory testing showed an elevation of inflammatory markers such as erythrocyte sedimentation rate (ESR) at 72 millimeters per hour (mm/h); C-reactive protein at 144.2 mg/L; normocytic normochromic anemia with hemoglobin (Hgb) at 9.8 g/dL; hematocrit at 30%; mean corpuscular volume (MCV) of 91.5 fL; mean corpuscular hemoglobin concentration (MCHC) at 32.7%. High-normal white blood count (WBC) at 10,500/mm^3^. Even though cellular differential was under normal limits, it was interesting that a slight increase of eosinophil relative mean was noticed during the first three days of hospital course ranging from 1.1% to 2.8%.

Urinalysis showed on repetitive occasions unspecific findings of moderate bacteria, moderate squamous epithelial cells, trace of blood with three red blood cells (RBCs) per high power field; leukocytes 10 white blood cells (WBCs) per high power field; and moderate positiveness for leukocyte esterase. Culture displayed with <10,000 colonies forming units (cfu)/ml of mixed gram-positive flora, and 10,000-50,000 cfu/ml of *Lactobacillus* species.

**Figure 3:**
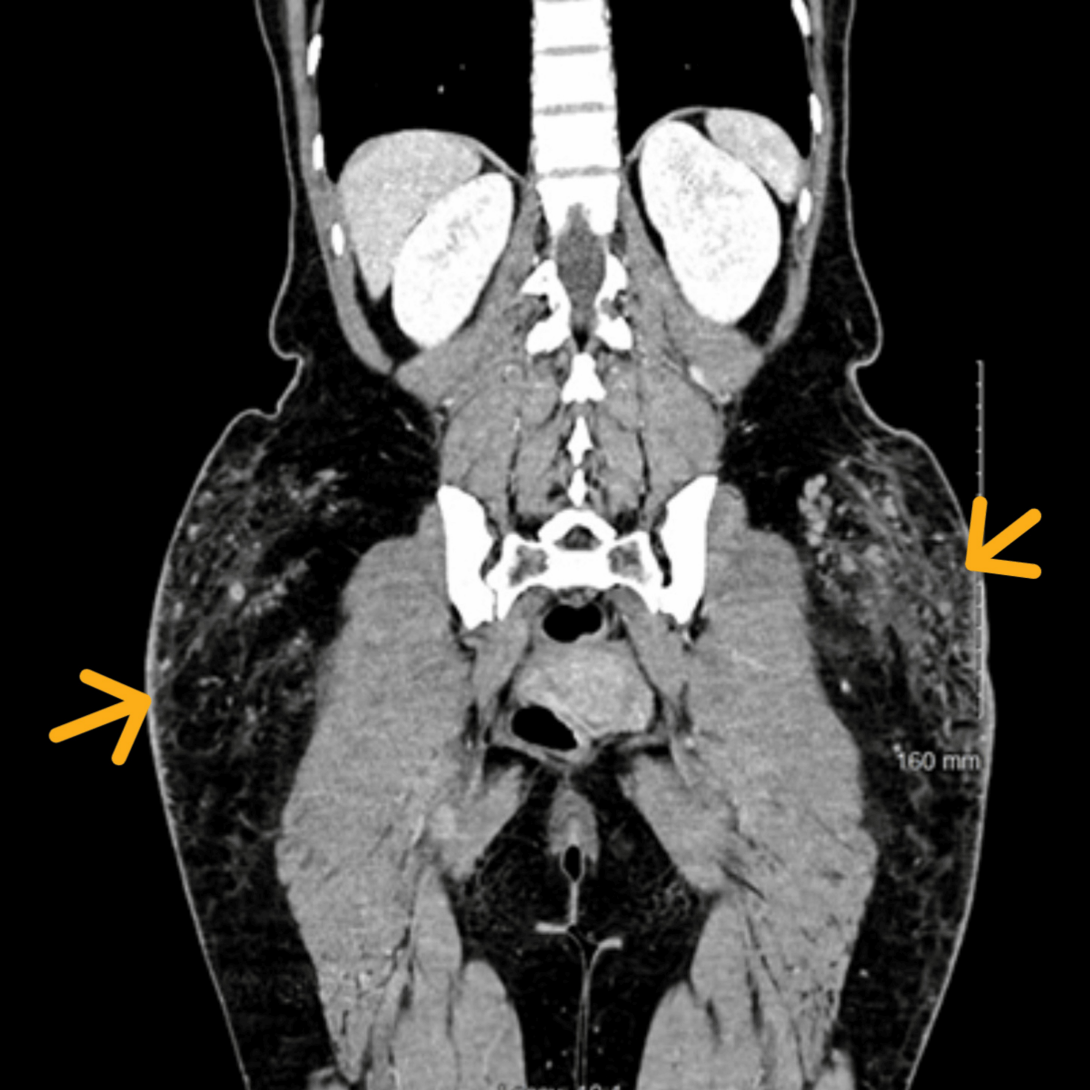
Coronal CT with contrast. Yellow arrows pointing at the extensive area of free material within the overlying connective tissue induration.

**Figure 4:**
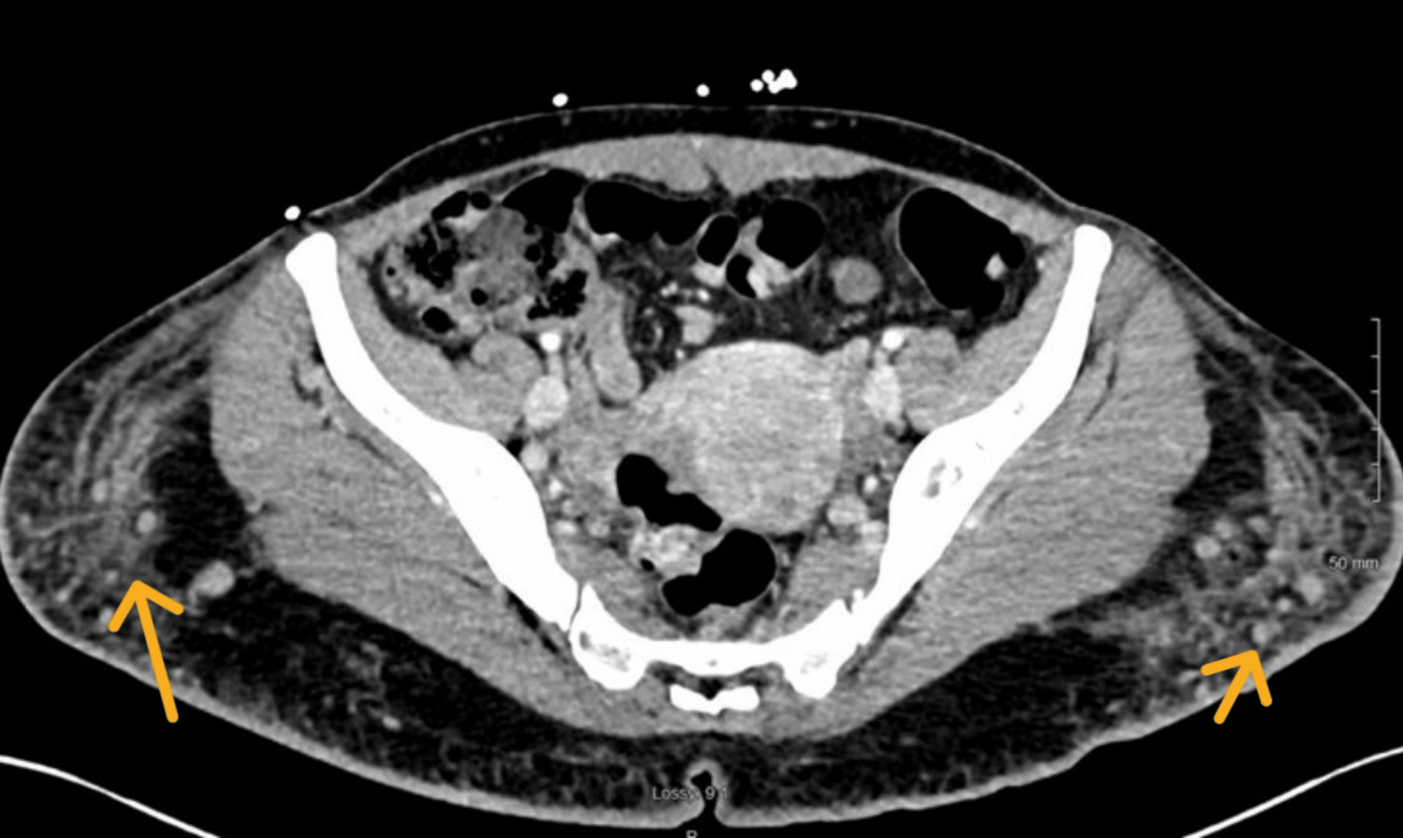
Axial CT with contrast. Yellow arrows pointing at free pellet-like findings suggestive of the injected substance.

Computed tomography (CT) scan of the abdomen and pelvis with contrast revealed extensive bilateral pellet-like findings suggestive of injected free substance similar to the appearance of free silicon, with overlying skin induration (Figures 3, 4).

Negative tests were also reported for screening and blood culture for methicillin-resistant *Staphylococcus aureus* (MRSA), human immunodeficiency virus antigen-antibody test (HIV AG-AB), antinuclear antibodies (ANA), and beta-human chorionic gonadotropin (beta- hCG) for pregnancy assessment.

Risk assessment for tuberculosis (Tb) was low, so testing was deferred. Complete metabolic panel showed no abnormalities. Electrocardiogram showed no conduction anomalies. Chest X-ray was unremarkable, with no suggestive findings of hilar adenopathy.

The patient was hospitalized for five days. During the first three days, she was initially given empiric antibiotic treatment for a suspected soft-tissue infection and a possible urinary tract infection (UTI). Given non-remission of symptoms and reconsideration of preliminary diagnoses, it was consensual that the urinary test results were likely a common finding for contamination. Additionally, a soft-tissue infection was doubtful because the inciting trigger occurred two years ago, and the patient did not exhibit any novel sign of an entry source for infection. Thus, after contemplating her current clinical status, her juvenile past medical history for sarcoidosis and no other conceivable origin for the ongoing chronic anemia, panniculitis due to erythema nodosum became the presumptive diagnosis and a 10-day course of steroid treatment with prednisone at 20 mg daily was started.

Upon consultation with the plastic surgeon, the patient was recommended against any urgent invasive procedure for biopsy sampling; instead, outpatient surgical follow-up for soft-tissue lesion and cervical lymphadenopathy were advised. In addition, it was not possible to perform such an intervention at the institution due to insurance coverage issues.

On the fifth day, the patient was discharged due to clinical and laboratory improvement under the diagnosis of panniculitis due to erythema nodosum as an extrapulmonary sarcoidosis presentation. Instructions were provided for the continuation of drug therapy at home and further follow-up appointments.

## Discussion

The most common form of panniculitis - aching condition of subcutaneous fat - is caused by erythema nodosum; at the same time, EN is the most common skin lesion in sarcoidosis. Erythema nodosum can be a recurrent chronic affection that is characterized by presenting in a bilateral manner with acute non-ulcerative signs and symptoms of inflammation. Patients often experience a prodromal phase of general malaise and constitutional symptoms, which may include the affecting of lymph nodes. Skin lesions may spontaneously resolve or may require pharmaceutical treatment either for symptomatic relief and/or to promote nodule involution, as demonstrated by the patient alleviation of clinical symptoms after the administration of steroids [2, 3].

Sarcoidosis is a non-caseating granulomatous immune disorder that is known to be a great imitator given that its variable presentations resemble different entities, misleading the diagnosis towards confounders. Even though only 2% of patients compound the cases without lung involvement, a subclinical course of a more disseminated granulomatous involvement from what is perceived from clinical history is suggested from post-mortem studies among sarcoidosis patients [4], hence the importance of preventing or limiting further extension of the disease by minimizing the exposure to triggers that have the potential to induce flares.

Currently, literature offers guidance regarding patients with the past medical history of sarcoidosis. Recommendation against dermal filler use is widely described, as its application carries the likelihood of triggering a flare in a predisposed subject, especially when the substance is a mixture of different biological and non-biological materials with variable antigenic strength and an unappropriated technique can injure the tissue by provoking inflammation or facilitating substance migration [5, 6].

Accordingly, professionals in this matter maintain that if fillers can induce granuloma formation in patients without sarcoidosis, it is undoubtedly not safe for patients with a stabilized disease [7]. Interestingly, it has been also described that signs and symptoms of sarcoidosis can be expressed in a wide range of time, between a few months and more than 30 years, after exposure to dermal fillers such as hyaluronic acid injections [6].

There is no doubt that capable healthcare professionals are the ones who would hold the best standard of care regarding guidance on individual medical decisions. However, the alarming increase in demand for cosmetic procedures that take place outside the regulatory protocol of a medical office has been driven by a vicious cycle of blinded consumer motivation for affordable and practical procedures, acquiescing to the offer from non-qualified personnel for cosmetic procedure services [8].

Even though some studies have proven that patients have a preference to be treated by nurses or physician assistants, most of the procedures are performed by aestheticians; this phenomenon illustrates the concern on the possibility that patients are resourceless to understand the difference among the variety of non-physician providers and their diverse levels of expertise for each treatment or procedure in a personalized manner [8].

In the case presented, the patient reported during the interview session that she was not asked about any relevant medical history by the cosmetic personnel she attended to. Also, she admitted a feeling that “something was wrong” during her cosmetic intervention, as she was unable to verify the practitioner’s credentials. She was also told that she would receive a superficial application of hyaluronic acid, but she couldn’t confirm whether that was the real content in the vial. Most concerning, she was astonished when the injections were administered profoundly into her tissue, rather than superficially on her stretch marks as she had been originally promised.

## Conclusions

Although extrapulmonary sarcoidosis is uncommon, patients with a past medical history of this condition might remain in remission for years until they meet a potential trigger that induces a hypersensitivity reaction, which can lead to localized granulomatous inflammation as is the case of erythema nodosum. For this reason, the authors of this article conclude that it is imperative for clinicians to explore further etiologic conditions when encountering dead ends in non-responsive patients to a particular therapy or clinical approach. The results of a good interview can certainly unravel the missing piece of a diagnostic puzzle and reorient the clinical approach.

On the other hand, patients are encouraged to seek board-certified and well-experienced practitioners when considering cosmetic interventions, especially those patients with particular medical conditions that can worsen when non-qualified personnel who lack the ability to collect a comprehensive and individualized clinical history of their patients/clients can potentially overlook the risks and side effects related to the treatments offered.
